# Prevalence of obsessive-compulsive disorders (OCD) symptoms among health care workers in COVID-19 pandemic: a systematic review and meta-analysis

**DOI:** 10.1186/s12888-023-05353-z

**Published:** 2023-11-21

**Authors:** Neda SoleimanvandiAzar, Ali Amirkafi, Mohammadreza Shalbafan, Seyyed Amir Yasin Ahmadi, Shadi Asadzandi, Shiva Shakeri, Mahdieh Saeidi, Reza Panahi, Marzieh Nojomi

**Affiliations:** 1 Preventive Medicine and Public Health Research Center, Psychosocial Health Research Institute, Department of Community and Family Medicine, School of Medicine, Iran University of Medical Sciences, Tehran, Iran; 2 Mental Health Research Center, Psychosocial Health Research Institute, Department of Psychiatry, School of Medicine, Iran University of Medical Sciences, Tehran, Iran; 3 Department of Medical Library and Information Science, School of Health Management and Information Sciences, Iran University of Medical Sciences, Tehran, Iran; 4 Research Center for Addiction and Risky Behaviors, Iran University of Medical Sciences, Tehran, Iran; 5 Department of Sociology and Anthropology, Nipissing University, North Bay, ON Canada

**Keywords:** OCD, Obsessive compulsive disorder symptoms, Health care workers, COVID-19, Systematic reviews, Meta-analysis

## Abstract

**Background:**

Obsessive Compulsive Disorder (OCD) symptoms, are among the serious mental health challenges that Health Care Workers (HCWs) faced during the COVID-19 pandemic. As these symptoms reduce the mental well-being and effectiveness of HCWs which are followed by poor health outcomes for patients, the aim of this systematic review and meta-analysis was to determine the prevalence of OCD symptoms among HCWs worldwide.

**Methods:**

PubMed, Google Scholar, Cochrane, Scopus, Web of Science, ProQuest, Emerald, and ERIC databases were searched using related keywords till the end of October 2021. Observational studies about the prevalence of OCD symptoms among healthcare workers during the COVID-19 pandemic were screened and evaluated. In order to assess the quality of studies, the Newcastle-Ottawa scale (NOS) checklist was used. The effect measure was the prevalence rate with a 95% confidence interval (CI).

**Results:**

A total of 7864 individuals from 11 studies were included. The range of OCD symptoms prevalence across these studies was from 0.07 to 0.47. Due to the high heterogeneity between the studies (*I*^*2*^ = 98.6%, *P* < 0.01), the random effects model was used. The pooled prevalence was 0.29 (95% CI: 0.22–0.38) based on logit transformed CI.

**Conclusions:**

The pooled prevalence of OCD symptoms was 29% among the HCWs during the COVID-19 pandemic. This prevalence was higher than the general population according to the pre-pandemic literature, but lower than the recent reports amid the pandemic. Psychosocial interventions are suggested to be designed and implemented in such conditions.

**Supplementary Information:**

The online version contains supplementary material available at 10.1186/s12888-023-05353-z.

## Introduction

The COVID-19 pandemic impacted different aspects of people’s lives; in addition to the economic and social effects, it also had a huge impact on health [1–3]. In addition to the physical burden, the mental health of the people has been wildly affected since the start of the outbreak; this includes increased depression, anxiety, obsessive-compulsive disorder (OCD), and post-traumatic stress disorder, plus worsening the psychiatric symptoms [4, 5].

OCD is one of the psychiatric disorders which was hugely impacted by the pandemic [6, 7]. During the pandemic, people faced various stressors and unexpected situations which were totally confusing; uncertainty, fear of contamination, lockdowns, strict public health measures, and hygiene protocols which involved social distancing and continuous hand washing made individuals redefine the new norm. The pandemic waves had such an unfavorable fingerprint that even by easing the COVID-19 restrictions, societies faced mental health inequalities and profound long-term consequences [8, 9]. Individuals with obsessive-compulsive traits may struggle more with adjusting to the easing of COVID-19 restrictions; they experienced exacerbation in symptoms of anxiety, fear, and tension when restrictions lifted, compared to the ones who didn’t have obsessive-compulsive traits beforehand [10]. These negative impacts could cause or worsen the OCD symptoms, whether through direct obsessive-compulsive behavior or indirectly as a stressor [11]. As a result, studies showed an increase in OCD symptoms among various groups, cultures and nations [12]. Among them, healthcare workers (HCWs) widely impacted.

Even before the pandemic, HCWs were prone to many mental health issues because of their stressful working conditions, violence and bullying [13, 14]; during the SARS and Influenza H1N1 pandemics, these issues were present and even escalated further for HCWs [15]. Throughout the COVID-19 pandemic, HCWs were in the frontline of the response; in addition to tolerating same stressors as the general public, long working hours in stressful situations and seeing the death of people and colleagues, had its toll on them physically and mentally [16, 17]. These symptoms were more severe among the less-experienced workers without enough social support and resiliency [18]. From another perspective, the daily protocols for HCWs consisted of repetitive sanitizing activities which exacerbated OCD symptoms; obsession with contamination and using protective equipment in addition to the compulsion for hygiene and the use of disinfectants are some instances [19, 20].

As the mental health of healthcare staff directly relates to better health outcomes for patients and fewer medical errors, it is necessary to assess and address their mental health issues comprehensively [21]; in particular, their OCD symptoms. There are some systematic reviews investigating OCD symptoms during the COVID-19 pandemic; one of them assessed the general population [6] and one focused on young people [22]. However, despite the importance of HCW’s mental health in the treatment process, there were no systematic studies investigating the effect of the COVID-19 pandemic on their OCD symptoms; therefore, in contrast to other studies, we aim to focus on HCWs and to find the prevalence of OCD symptoms among them.

## Methods

We followed Preferred Reporting Items for Systematic Reviews and Meta-Analyses (PRISMA) statement by the Cochrane Collaboration [23] to conduct this review.

### Search strategy

We conducted an advanced search in PubMed, Google Scholar, Cochrane, Scopus, Web of Science, ProQuest, Emerald and ERIC databases (Table 1). We performed the search on October 25th, 2021. The keywords, based on Medical Subject Headings (MeSH), were retrieved; an example of search query used to retrieve the papers in Web of Science is as follows: TS=( ( disorder AND “Obsessive-Compulsive” OR disorders AND “Obsessive-Compulsive” OR “Obsessive-Compulsive Disorders” OR neurosis AND “Obsessive-Compulsive” OR neuroses AND “Obsessive-Compulsive” OR “Obsessive-Compulsive Neurosis” OR " Anankastic Personalities” OR “Obsessive compulsive symptoms” OR OCD) AND ( COVID-19 OR “2019 Novel Coronavirus Disease” OR “2019 Novel Coronavirus Infection” OR “2019-nCoV Disease” OR “2019-nCoV Infection” OR “COVID-19 Pandemics " OR “COVID-19 Virus Disease” OR “COVID-19 Virus Infection” OR “Coronavirus Disease 2019” OR “Coronavirus Disease-19” OR “SARS Coronavirus 2 Infection” OR “SARS-CoV-2 Infection”) AND (“Health Personnel” OR “Health Care Professionals” OR “Health Care Provider” OR “Healthcare Providers” OR “Healthcare Workers”)). After retrieving the studies, duplicated ones were excluded using Endnote X8.2.

**Table 1:** Search results based on databases

Database	Number of records
ERIC	200
PubMed	706
Scopus	292
Web of Science	118
Google Scholar	30
ProQuest	100
Emerald	468
Cochrane	37
Total	1951
Duplicates	163
Total without duplicates	1788

### Inclusion criteria

The inclusion criteria for this review were: (1) be in English language, (2) be original and peer reviewed (3) be consistent with the purpose of the research, (4) be published until the end of October 2021, and (5) availability of articles’ full texts. These criteria were taken into consideration by the researchers during the screening of the title and abstract of the studies as well as the articles’ full text in order to retrieve eligible articles.

### Screening process, critical appraisal and data extraction

Firstly, the titles and abstracts of all articles were screened by two independent researchers based on the inclusion criteria to exclude the irrelevant ones. Afterwards, two authors performed the same screening on the remaining articles’ full-texts independently. During these processes, all inconsistencies between the reviewers were resolved through re-assessment of the issue by a third researcher; final decision was made by a consensus among three of them.

In the next step, we extracted data from each of the eligible articles, including title, name(s) of author(s), publication place and year, research sample or population, type of study, objectives, key findings and results. We considered PRISMA diagram for assessing the retrieval, extraction and removal of the articles. Quality assessments were independently conducted by two authors, and in the case of disagreement, the studies were referred to a third researcher. Newcastle-Ottawa scale (NOS) for cross-sectional studies was used in this regard. If a study got a score higher than 6, it was considered to have a good quality, a 5–6 score was categorized as satisfactory, and lower than 5 score was considered as unsatisfactory quality [24].

### Data synthesis and analysis

The prevalence rates (as the effect measure) were synthesized using logit transformed error estimation, and 95% confidence intervals (CI) were reported. Common and random effects models were used to estimate the pooled effect. I^2^ was reported for heterogeneity and funnel plot to visualize publication bias. Heat map was also used for investigation of other variables (in t-score) at the level of individual studies. Pairwise comparison of the demographic variables was carried out by correlation matrix. All the statistical procedures were performed in *R version 4.2.1* (R foundation for statistical computing, Austria) software, *Meta* package.

## Results

### Study selection

**Fig. 1:**
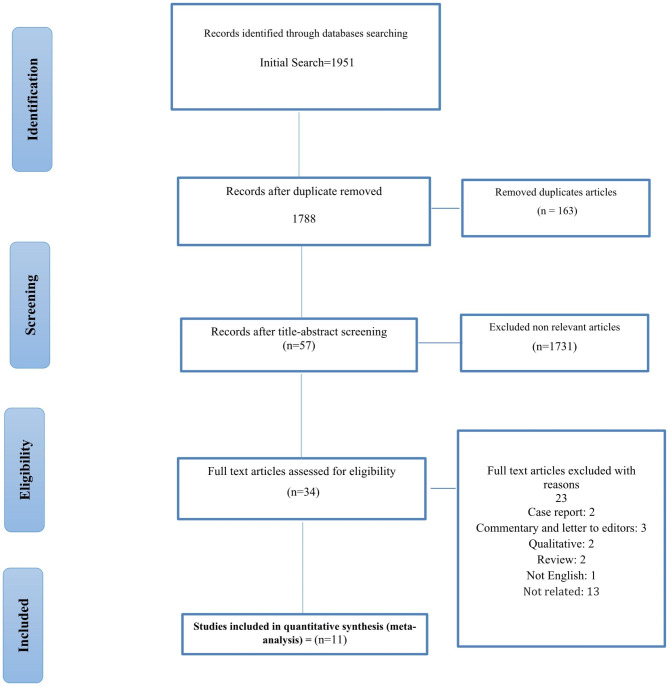
PRISMA 2009 flow diagram

After duplicate removal, 1788 titles and abstracts of unique articles were screened. 34 full-text articles were assessed for eligibility; among them, 23 articles were excluded. In total, 11 studies [25–35] involving 7864 participants in 5 countries were included in the meta-analysis of Prevalence of OCD symptoms among HCWs during COVID‑19 Pandemic and their data was extracted (PRISMA Flow diagram in Fig. 1).

### Risk of bias assessment

Quality assessment was conducted following the data extraction, using the NOS checklist for cross-sectional studies [24]; this checklist is consisted of three sections; “selection” which is consisted of four questions, “comparability” consisted of one question and “outcome”, consisted of two questions. According to these questions, the result of quality assessment is as follows; for the “selection” part: Q1) three studies used random sampling, while the others failed to get its point; Q2) three studies did not calculate sample size or their sample size was small, while the other studies calculated the sample size or their sample size was large enough; Q3) according to the topic of studies, it was logically expected that response rates were enough and no concern was observed in the full texts in this regard; Q4) ascertainment of the exposure (being healthcare worker and working during the pandemics) was logically valid in all the studies based on the official and legal positions of the individual participants in their hospitals. For “comparability” section, all studies failed to get any points as they were descriptive, and no variable adjustment was conducted. For the “outcome” section: Q1) the measure of outcome was objective and valid in all the studies as they used verified questionnaires for detection of OCD symptoms; Q2) all the studies failed to get this point as no statistical test was applicable.

**Fig. 2:**
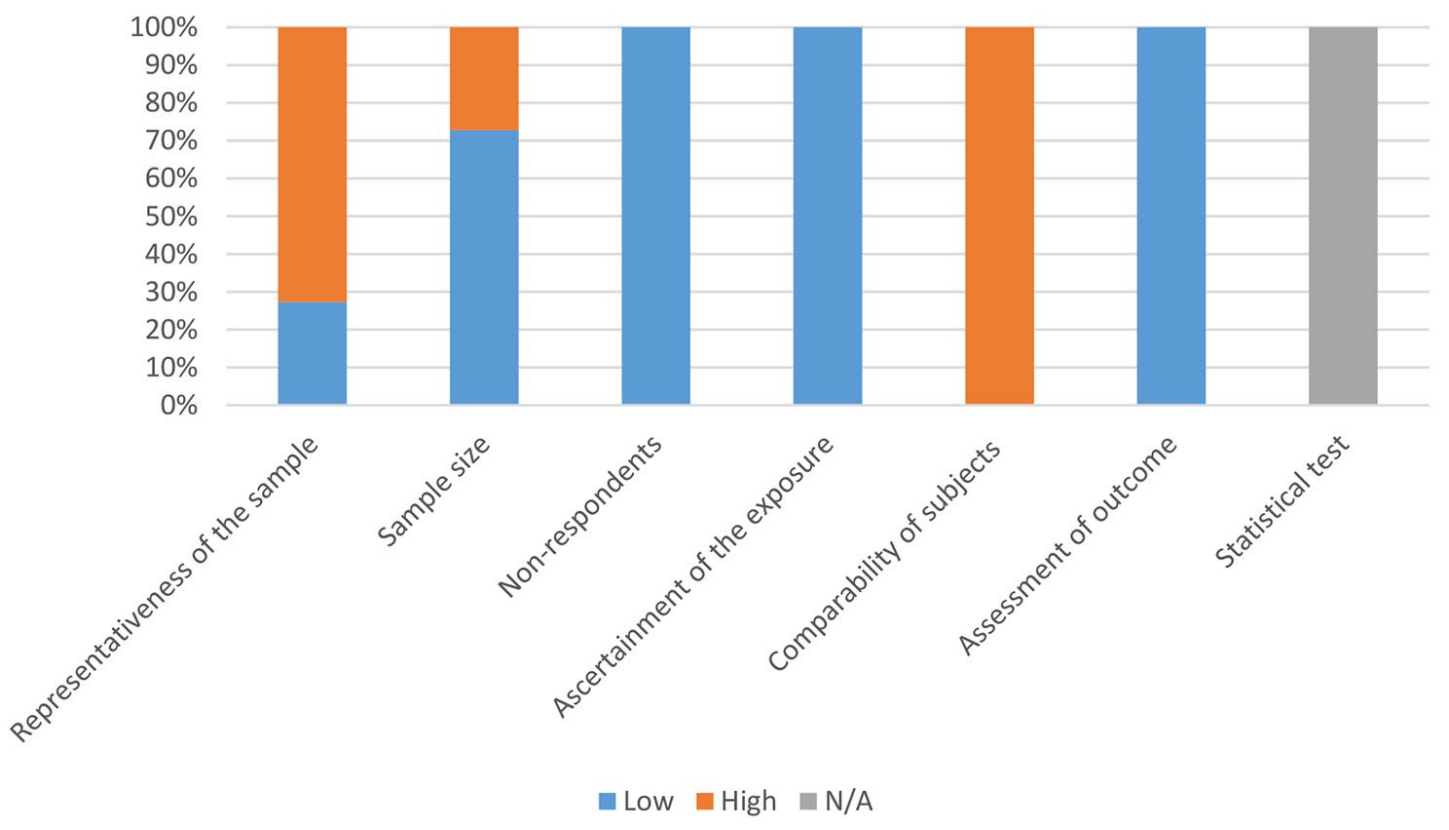
Risk of bias assessment based on NOS checklist

As a verdict, no study was excluded because of poor quality, three studies had good quality and quality of eight was satisfactory. The results of NOS evaluation are shown in Fig. 2.

### Results of individual studies

Sample size and location of the primary studies in addition to the characteristics of the participants are summarized in Table 2. In total, the results of 7864 participants from 11 studies from Canada, China, Egypt, Iraq and Spain were synthesized. The range of event rates (prevalence) was from 0.07 to 0.47 while the mean age range was from 20.73 to 42.79 years; the range of male participant percentage was from 0 to 61.93% and the range of single participants’ percentage was from 0 to 96.47%.

**Table 2:** Background information of individual studies

Study	Year	Country	Age mean (year) ^1^	Male (%)	Single (%)	Event	Sample size	Instrument	Quality score^4^
Taqi Mohammed Jwad Taher [32]	2021	Iraq	20.73	32.12	96.47	707	1644	OCI-R	6
Jun Xing [33]	2020	China	36.77	27.92	25.73	204	548	SCL-90	6
Wen-rui Zhang [34]	2020	China	40.16	35.72	17.97	154	2182	SCL-90-R	6
Yahua Zheng [35]	2021	China	40.45	15.47	25.73 ^3^	53	207	SCL-90	5
Mohamed Abdelghani [25]	2021	Egypt	39.5	61.93	11.93	45	218	SCL-90-R	7
Gellan K. Ahmed [26]	2021	Egypt	34.26	40.98	24.95	36	122	Y-BOCS	6
Yang Juan [27]	2020	China	31.16	29.39	46.93	171	456	Y-BOCS	7
Min Liu ^2^ [28]	2021	China	29.8	0	0	19	83	SCL-90	5
Kelly Mrklas [29]	2020	Canada	42.79	7.4	12.94	663	1414	BOCS	7
Xiuli Ou [30]	2021	China	30.48	7.6	51.09	27	92	SCL-90	5
Sergio Reno-Chanca [31]	2021	Spain	41.13	28.9	25.73 ^3^	337	898	Y-BOCS	6

(1) In some studies, mean age was indirectly calculated from their interval reports (if lack of reporting the range, the minimum and maximum ages were regarded as 18 and 70, respectively). (2) This study consisted of pregnant women. (3) This variable was missing in these two studies. Therefore, the median of other studies was replaced. (4) Based on NOS checklist for cross-sectional studies (0–4: unsatisfactory, 5–6: satisfactory, 7–8: good, 9–10: very good)

OCI-R: Obsessive-Compulsive Inventory-Revised. SCL-90: Symptom Checklist-90. SCL-90-R: SCL-90 revised. Y-BOCS: Yale–Brown Obsessive–Compulsive Scale. BOCS: Brief Obsessive-Compulsive Scale

### Results of synthesis

**Fig. 3:**
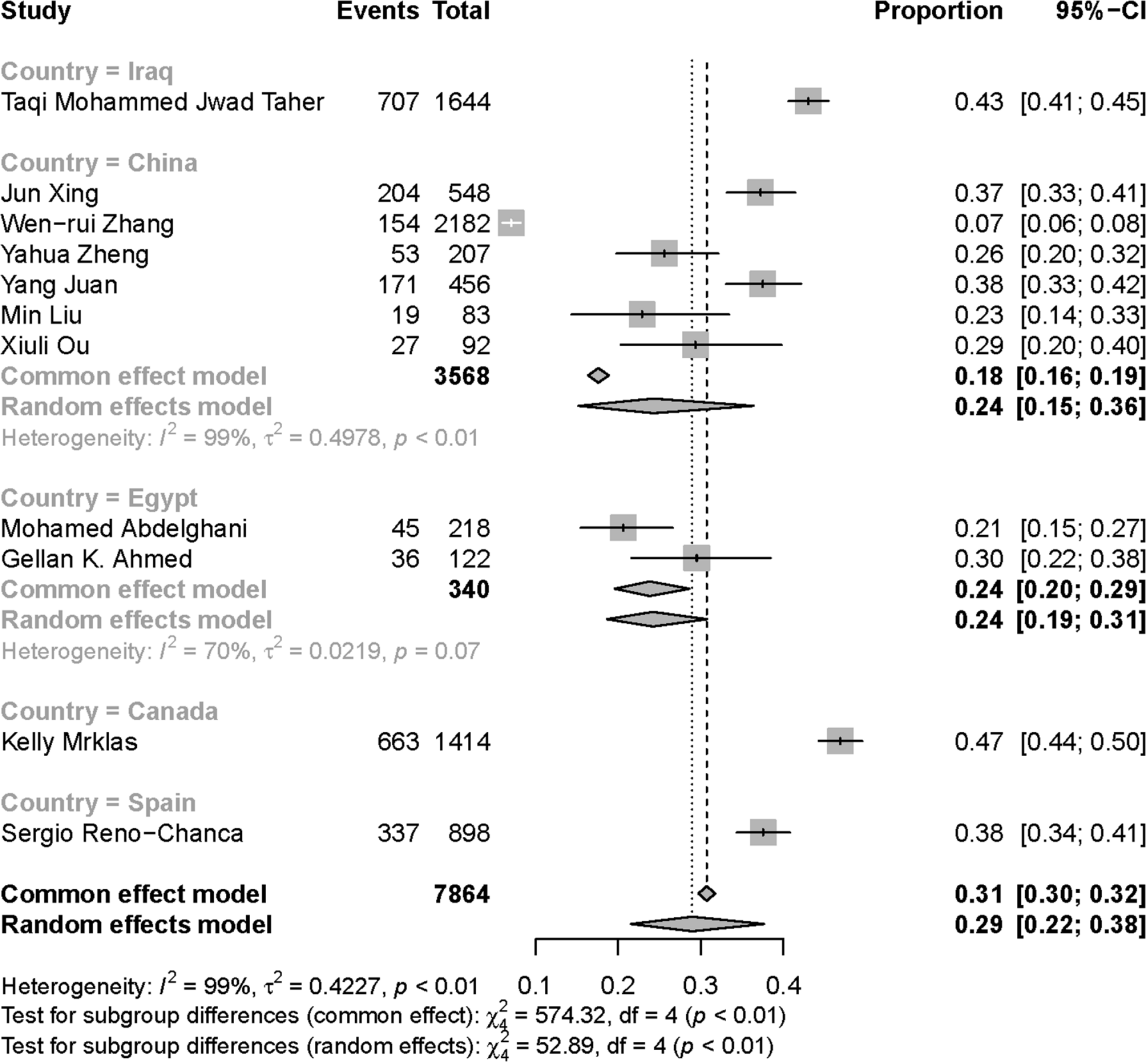
Forest plot of the prevalence rates. Random effects model was considered

**Fig. 4:**
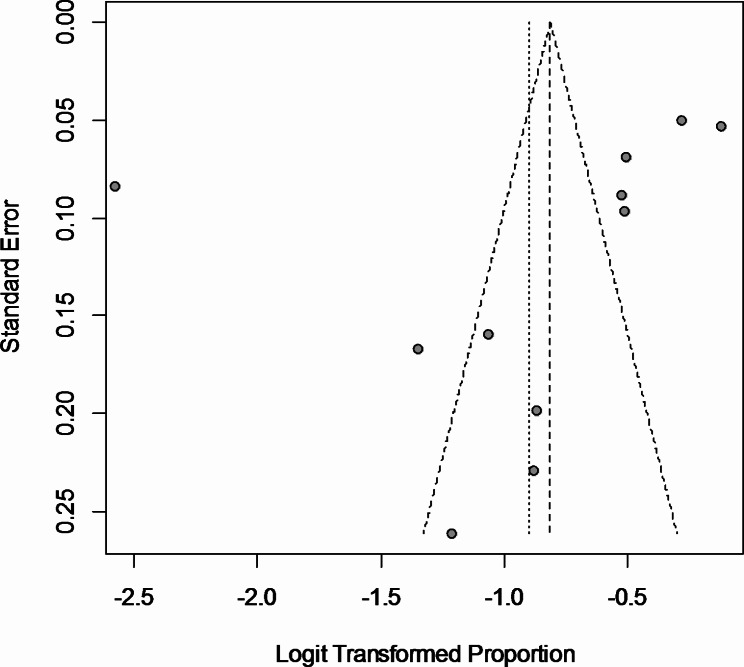
Funnel plot of the prevalence rates

**Fig. 5:**
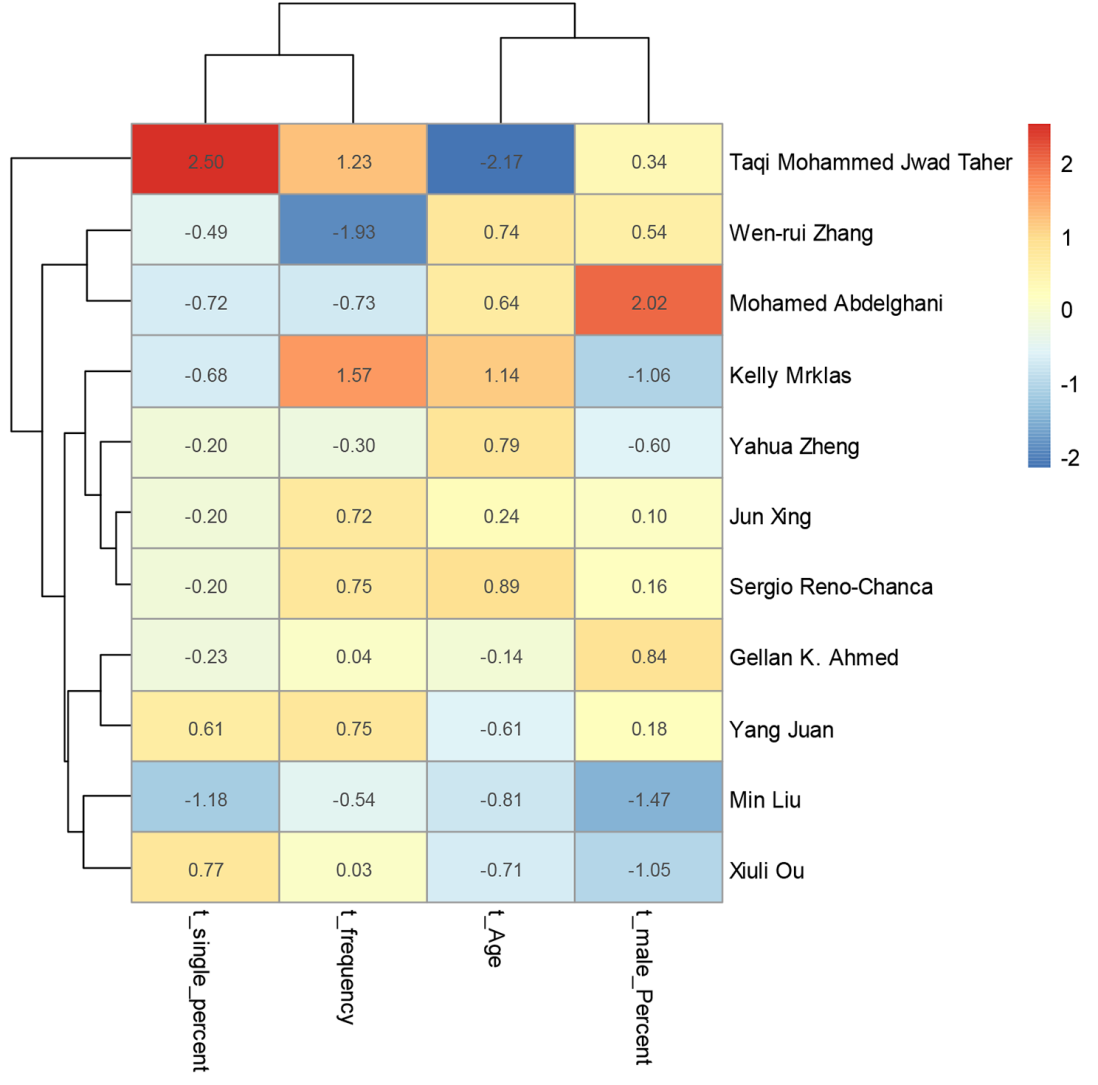
Heatmap for comparison of individual studies-based t-scores of their demographic variables. T-score of frequency (event rate) was calculated based on random effects model pooled rate (29%) while t-score of other variables were calculated based on simple means

**Fig. 6:**
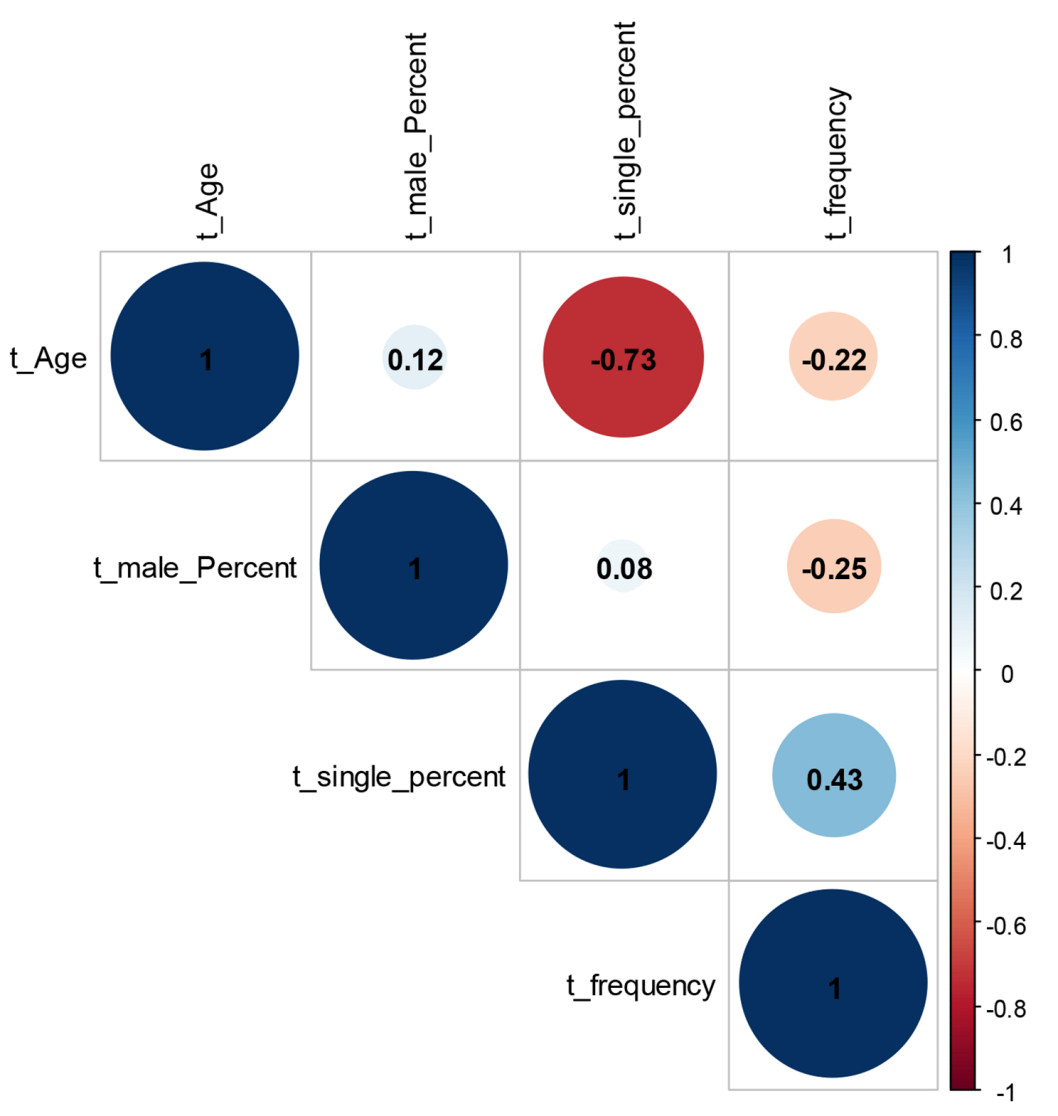
Correlation plot based on Pearson correlation matrix. Because of small sample size (n = 11) and unweighted analysis, P values were not reported

Due to the high heterogeneity of the effect sizes (*I*^*2*^ = 98.6%, *P* < 0.01), random effects model was implemented. Accordingly, the pooled prevalence was 0.290 (95% CI: 0.216–0.377). Subgroup analysis based on countries showed a significant difference between the countries (*P* < 0.01, random effects) in which China showed the least prevalence rate. The forest plot of overall and subgroup analysis is shown in Fig. 3. The funnel plot showed an asymmetric distribution of the effect sizes (Fig. 4). The heterogeneity of the individual studies is also shown visually. According to the heat map, the study of Taqi Mohammed Jwad Taher showed the strongest source heterogeneity as it had the highest percentage of singles and the lowest age (Fig. 5). No strong correlation was found between OCD symptoms frequency and the demographic variables (*r* < 0.7). The highest correlation in this regard was for the correlation of prevalence with percentage of singles (*r* = 0.43) (Fig. 6).

No significant predictor was observed among the demographic variables based on multiple and simple meta-regression modeling other than the differences of countries. The covariates were age, year, percentage of males and percentage of singles. In the multiple regression modeling, the most significant association was for percentage of singles (*P* = 0.135), and in the simple regression modeling, again this variable showed the most significant association (*P* = 0.167). Sensitivity analysis was conducted with the scenario of comparing the pooled results of good quality studies with total studies. Accordingly, despite the fact that the good quality studies showed a more pooled prevalence rate (0.34, 95% CI: 0.23–0.48, random effects), this pooled effect (0.34) was covered by the 95% CI of the total studies’ pooled effect (0.22–0.38, random effects). In addition, the meta-regression of NOS score did not show a significant impact (*P* = 0.438).

## Discussion

To the best of our knowledge, this study represents the first systematic review and meta-analysis aimed at reporting the prevalence of OCD symptoms among HCWs amid the COVID-19 pandemic. The main finding implies that the mean prevalence of OCD symptoms among HCWs is 29%.

Many studies have estimated the prevalence of OCD worldwide; the lifetime prevalence of OCD has been estimated at around 2.5% globally and 2.8% in Eastern Mediterranean Region [36]. In contrast, the prevalence of OCD symptoms has been less studies, particularly in Iran; one the few publications, estimated the prevalence of OCD symptoms at 11.2% in high school adolescents [37]. Data on the prevalence of OCD and OCD symptoms among HCWs is even scarcer; one study have reported that the prevalence of minor mental disorders including OCD in healthcare workers was 22%, compared to 17% among normal control groups from general population, before the pandemic [38]. Notably, this estimate may vary depending on the studied populations and the assessment tools used.

Since the beginning of the COVID-19 outbreak, this trend changed and several studies evaluated the impact of the special circumstances of the pandemic on the mental health of both general population and healthcare workers. Most of these studies focused on anxiety, stress and depression. Among limited studies investigating OCD symptoms, most claimed that the pandemic had caused a significant increase in OCD symptoms in patients who already suffered from OCD and related disorders [39, 40]. For instance, Khosravani et al. (2021) noted that OCD symptom scores and general OCD severity in patients with OCD were significantly higher during the COVID-19 pandemic compared to the pre-pandemic period; they also suggested that the increase in symptoms and severity might be due to the mental pressure which was induced by the pandemic [41]. Tanir et al. (2020) also claimed that OCD symptoms have worsened in patients during the pandemic. Contamination obsessions and cleaning/washing compulsions were the most frequent OCD symptoms even before the pandemic, and their frequency increased as the pandemic occurred [20].

We could not find any literature about the OCD prevalence among healthcare workers prior to the pandemic for comparison with present results. Although there were some studies that compared the OCD prevalence among HCWs and general population, they didn’t report convergent findings. Zhang et al. (2020) stated that medical health workers had a significantly higher prevalence of OCD (5.3%) compared to non-medical health workers (2.2%). They also had higher scores of OCD symptoms [34]. Reno-Chanca et al. (2021) found that HCWs had higher scores of OCD symptoms compared to the general population [31]. In another study by Ergenc et al. (2020), healthcare workers in the COVID-19 section had significantly more OCD symptoms, compared to the control group who had worked in non-COVID Sect. [42]. On the other hand, a recent systematic review and meta-analysis by Jalalifar et al. reported that the overall prevalence of OCD symptoms among the general population amid the pandemic was 41.2%. Despite reporting a higher percentage compared to our study (29%), they concluded that being a hospital staff may be a predictor of OCD symptoms among the general population [43]. As it is shown, the interpretation of our findings in comparison with previous studies is not conclusive to answer, whether the prevalence of OCD among HCWs, as a whole, is higher than the general population or not.

It can be expected that HCWs are more prone to OCD; as they have more exposure and risk of infection; direct contact with COVID-19 patients, strict protection and safety instructions in the hospital environment, severe emphasis on hand-washing and disinfecting personal equipment, observing colleagues getting infected with the virus, and the fear of being infected or spreading the disease to their families or relatives are among the reasons [44]. Nevertheless, resilience and initiatives to improve mental health, could explain lower prevalence of OCD symptoms among HCWs in comparison to the general population [45].

No predictor was found for OCD prevalence based on regression and correlation analyses. This finding is in line with recent meta-analysis of Jalalifar et al. which shows the prevalence of OCD symptoms was insignificantly higher among females in general population during the COVID-19 pandemic [43].

The pooled results of subgroups (countries) indicates that the pooled prevalence rates in China and Egypt were lower than the overall pooled prevalence (24% vs. 29%), but this difference was not significant based on 95% CI; other countries had higher prevalence rates than the overall pooled prevalence. Therefore, countries with multiple studies showed less pooled prevalence rate, while countries with a single study showed more prevalence rate. This might be due to publication bias for countries with a single study. Cultural aspects also have a key role in this difference; as it has been shown by previous studies that the prevalence of OCD before and during the pandemic varies from country to country. In this way, Jalalifar et al. meta-analysis also reported that studies in the Middle East show a higher prevalence of OCD symptoms among the general population [43]. Due to limited number of studies which include only 5 countries, we are not able to conclude this fact among HCWs.

The other subgroup analysis – which was conducted as a sensitivity analysis – showed a more pooled prevalence rate in good quality studies vs. total (34% vs. 29%); however, this difference was not significant based on 95% CI. This finding shows that the exclusion of lower-quality studies could not reduce the pooled prevalence rate.

The prevalence rates were heterogenic in a wide range. This heterogeneity might be due to different methods of symptom evaluation. One of the methodological differences that can influence results was the use of different psychometric tools for the measurement of prevalence. Three studies used Yale-Brown Obsessive–Compulsive Scale, one study used Obsessive-Compulsive Inventory-Revised and another one used Brief Obsessive-Compulsive Scale; all three instruments assess OCD symptoms severity but each has its own strengths and weaknesses. The rest of the studies used the Symptom Checklist-90 which has a subscale for OCD symptoms; however, it has less reliability compared to other mentioned instruments [46]. In addition, none of these tolls are specific for COVID-19 [47].

According to the heat map, the most variation in demographic characteristics was for the study of Taher et al. (2021) in Iraq; in which more cases were young and single, and had the second highest prevalence of OCD symptoms which was 43% [32]. Therefore, this study was considered as the most important source of heterogeneity. The asymmetric distribution of the funnel plot showed a potential publication bias which is probable in these topics. According to this funnel plot, larger studies showed higher prevalence rates. As mentioned before, the source of this potential risk of publication bias might be attributed to the countries with a single study (i.e., Iraq, Spain, and Canada); because a single study might be affected by selection bias, while repetitive studies may neutralize this limitation.

### Strengths and limitations of the study

The present study reports the pooled prevalence of OCD among HCWs during COVID-19 pandemic. Naturally, this study was not immune to limitations. The most important limitation was the high heterogeneity and a potential publication bias; using the random effects model helped us reduce the influence of heterogeneity. For the concern of publication bias, the funnel plot showed a lower prevalence in smaller studies and a higher prevalence in larger studies. It seems that completing the gaps of the funnel plot, results in similar pooled prevalence. In terms of quality assessment, the most important source of bias was the representativeness of the samples. Hence, another important limitation was the non-probability sampling in many studies. Other than these technical limitations, there were some thematic and generic limitations; (1) there was no comparison between pre-COVID-19, COVID-19, and post-COVID-19 era due to the scarcity of available data; (2) effect size changes were probable during different waves of the pandemic; (3) there were differences among the methods and tools for OCD symptom recognition, and (4) lack of instruments to distinct self-care from obsessive behaviors.

## Conclusion

The pooled prevalence of OCD symptoms was 29% among the HCWs during the COVID-19 pandemic. This prevalence was higher than the general population according to the pre-pandemic literature, but lower than the recent reports amid the pandemic.

As poor mental health of HCWs leads to poor treatment outcomes for the patients, improving their mental health is imperative. Thus, proper interventions and activities aimed at increasing mental health well-being and reducing OCD symptoms among HCWs should be integrated into the workplace schedule. Also, constant monitoring and on-time psychological interventions must be implemented.

### Electronic supplementary material

Below is the link to the electronic supplementary material.

Supplementary Material 1

## Data Availability

All data generated or analyzed during the current study are available from the corresponding author on reasonable request.

## List of abbreviations

OCD: Obsessive Compulsive Disorder
HCWs: Health Care Workers
PRISMA: Preferred Reporting Items for Systematic Reviews and Meta-Analyses
NOS: Newcastle-Ottawa scale
