# Supplementary material for: Prevalence of obsessive-compulsive disorders (OCD) symptoms among health care workers in COVID-19 pandemic: a systematic review and meta-analysis
Source: BMC Psychiatry. 2023 Nov 21;23:862. doi: 10.1186/s12888-023-05353-z (PMC10664495; doi:10.1186/s12888-023-05353-z)
Supplement: Supplementary file 1 — Supplementary Material 1 [file 12888_2023_5353_MOESM1_ESM.docx]

| **DATE** | **NUMBER** | **SEARCH STRATEGY** | **DATABASE** | **ROW** |
| --- | --- | --- | --- | --- |
| 25-10-2021 | 118 | TS=( ( disorder AND "Obsessive-Compulsive" OR disorders AND "Obsessive-Compulsive" OR "Obsessive-Compulsive Disorders" OR neurosis AND "Obsessive-Compulsive" OR neuroses AND "Obsessive-Compulsive" OR "Obsessive-Compulsive Neurosis" OR " Anankastic Personalities" OR "Obsessive compulsive symptoms" OR OCD ) AND ( covid-19 OR "2019 Novel Coronavirus Disease" OR "2019 Novel Coronavirus Infection" OR "2019-nCoV Disease" OR "2019-nCoV Infection" OR "COVID-19 Pandemics " OR "COVID-19 Virus Disease" OR "COVID-19 Virus Infection" OR "Coronavirus Disease 2019" OR "Coronavirus Disease-19" OR "SARS Coronavirus 2 Infection" OR "SARS-CoV-2 Infection") AND (“Health Personnel” OR “Health Care Professionals” OR “Health Care Provider” OR “Healthcare Providers” OR “Healthcare Workers”)) | **WOS** | 1 |
| 25-10-2021 | 292 | TITLE-ABS-KEY ( ( disorder AND "Obsessive-Compulsive" OR disorders AND "Obsessive-Compulsive" OR "Obsessive-Compulsive Disorders" OR neurosis AND "Obsessive-Compulsive" OR neuroses AND "Obsessive-Compulsive" OR "Obsessive-Compulsive Neurosis" OR " Anankastic Personalities" OR "Obsessive compulsive symptoms" OR OCD ) AND ( covid-19 OR "2019 Novel Coronavirus Disease" OR "2019 Novel Coronavirus Infection" OR "2019-nCoV Disease" OR "2019-nCoV Infection" OR "COVID-19 Pandemics " OR "COVID-19 Virus Disease" OR "COVID-19 Virus Infection" OR "Coronavirus Disease 2019" OR "Coronavirus Disease-19" OR "SARS Coronavirus 2 Infection" OR "SARS-CoV-2 Infection") AND (“Health Personnel” OR “Health Care Professionals” OR “Health Care Provider” OR “Healthcare Providers” OR “Healthcare Workers”)) | **Scopus** | 2 |
| 25-10-2021 | 30 | ( ( disorder AND "Obsessive-Compulsive" OR disorders AND "Obsessive-Compulsive" OR "Obsessive-Compulsive Disorders" OR neurosis AND "Obsessive-Compulsive" OR neuroses AND "Obsessive-Compulsive" OR "Obsessive-Compulsive Neurosis" OR " Anankastic Personalities" OR "Obsessive compulsive symptoms" OR OCD ) AND ( covid-19 OR "2019 Novel Coronavirus Disease" OR "2019 Novel Coronavirus Infection" OR "2019-nCoV Disease" OR "2019-nCoV Infection" OR "COVID-19 Pandemics " OR "COVID-19 Virus Disease" OR "COVID-19 Virus Infection" OR "Coronavirus Disease 2019" OR "Coronavirus Disease-19" OR "SARS Coronavirus 2 Infection" OR "SARS-CoV-2 Infection")) | **Google** | 3 |
| 25-10-2021 | 706 | ((((((((((((((((("Obsessive-Compulsive"[Title/Abstract]) OR ("Obsessive-Compulsive Disorders"[Title/Abstract])) OR ("Obsessive-Compulsive Neurosis"[Title/Abstract])) OR (" Anankastic Personalities"[Title/Abstract])) OR ("Obsessive compulsive symptoms"[Title/Abstract])) OR (OCD[Title/Abstract])) AND (covid-19[Title/Abstract])) OR ("2019 Novel Coronavirus Disease"[Title/Abstract])) OR ("2019 Novel Coronavirus Infection"[Title/Abstract])) OR ("2019-nCoV Disease"[Title/Abstract])) OR ("2019-nCoV Infection"[Title/Abstract])) OR ("COVID-19 Pandemics "[Title/Abstract])) OR ("COVID-19 Virus Disease"[Title/Abstract])) OR ("COVID-19 Virus Infection"[Title/Abstract])) OR ("Coronavirus Disease 2019"[Title/Abstract])) OR ("Coronavirus Disease-19"[Title/Abstract])) OR ("SARS Coronavirus 2 Infection"[Title/Abstract])) OR ("SARS-CoV-2 Infection"[Title/Abstract]) | **PubMed** | 4 |
| 25-10-2021 | 468 | title:"Obsessive-Compulsive Disorders" OR (title:"Obsessive-Compulsive Neurosis") OR (title:"OCD") AND (title:"covid-19") OR (title:"2019-nCoV Disease") OR (title:"COVID-19 Pandemics ") OR (title:"COVID-19 Virus Disease") OR (title:"COVID-19 Virus Infection") OR (title:"SARS-CoV-2 Infection")  <https://www.emerald.com/insight/search?q=title%3A%22Obsessive-Compulsive+Disorders%22+OR+%28title%3A%22Obsessive-Compulsive+Neurosis%22%29+OR+%28title%3A%22OCD%22%29+AND+%28title%3A%22covid-19%22%29+OR+%28title%3A%222019-nCoV+Disease%22%29+OR+%28title%3A%22COVID-19+Pandemics+%22%29+OR+%28title%3A%22COVID-19+Virus+Disease%22%29+OR+%28title%3A%22COVID-19+Virus+Infection%22%29+OR+%28title%3A%22SARS-CoV-2+Infection%22%29&advanced=true&fromYear=2020&toYear=2021> | **Emerald** | 5 |
| 25-10-2021 | 37 | "Obsessive-Compulsive Disorders" in Title Abstract Keyword OR "Obsessive-Compulsive Neurosis" in Title Abstract Keyword OR OCD in Title Abstract Keyword AND covid-19 in Title Abstract Keyword OR "COVID-19 Pandemics " in Title Abstract Keyword - (Word variations have been searched) | **Cochrane** | 7 |
| 25-10-2021 | 100 | ti("Obsessive-Compulsive Disorders" ) OR ti("Obsessive-Compulsive Neurosis") OR ti("Obsessive compulsive symptoms" ) OR ti( " Anankastic Personalities" ) OR ti(OCD) AND ti( "COVID-19 Pandemics " ) OR ti("SARS Coronavirus 2 Infection") OR ti("COVID-19 Virus Infection") OR ti("2019 Novel Coronavirus Disease" ) OR ti(covid-19) | **ProQuest** | 9 |
| 25-10-2021 | 200 | ( ( disorder AND "Obsessive-Compulsive" OR disorders AND "Obsessive-Compulsive" OR "Obsessive-Compulsive Disorders" OR neurosis AND "Obsessive-Compulsive" OR neuroses AND "Obsessive-Compulsive" OR "Obsessive-Compulsive Neurosis" OR " Anankastic Personalities" OR "Obsessive compulsive symptoms" OR OCD ) AND ( covid-19 OR "2019 Novel Coronavirus Disease" OR "2019 Novel Coronavirus Infection" OR "2019-nCoV Disease" OR "2019-nCoV Infection" OR "COVID-19 Pandemics " OR "COVID-19 Virus Disease" OR "COVID-19 Virus Infection" OR "Coronavirus Disease 2019" OR "Coronavirus Disease-19" OR "SARS Coronavirus 2 Infection" OR "SARS-CoV-2 Infection")) | **ERIC** |  |

**Supplemantory Table 1- Search Syntax in the Scientific Databases.** **ERIC:** **Education Resources Information Center ;WOS:** **Web Of Science**
